# Victims of Violence and Post-Discharge Adverse Events: A Prospective Modified Trauma Quality Improvement Program (TQIP) Study

**DOI:** 10.7759/cureus.18630

**Published:** 2021-10-09

**Authors:** Brendan R Gontarz, Usman Siddiqui, Carol McGuiness, Andrew Doben, Vijay Jayaraman, Erin Mclaughlin, Stephanie Montgomery, Manuel Moutinho, David S Shapiro

**Affiliations:** 1 Surgery, University of Connecticut, Hartford, USA; 2 Surgery, Trinity Health of New England, Hartford, USA; 3 Trauma, Trinity Health of New England, Hartford, USA; 4 Surgery, Critical Care, Palliative Care & Trauma, Saint Francis Hospital (Trinity Health of New England), Hartford, USA

**Keywords:** post trauma, ed utilization, readmission risk, violence, tqip

## Abstract

Introduction

Trauma patients frequently return to an emergency department (ED) soon after discharge; often for non-urgent reasons. Social factors contribute to higher ED usage. At present, there is no standardized system for reporting of ED visits and readmissions among trauma care. We hypothesized that victims of violent crime suffer from many early post-discharge adverse events that has not been captured by current methods.

Methods

We prospectively consented and enrolled injured patients from January 1st, 2019 to December 31st, 2019. We documented 30-day post-discharge events using post-discharge phone calls and detailed chart abstraction. Patients were categorized as victims of violence (VV) or unintentional traumatic injury (UT).

Results

During the study period, 444 patients were enrolled. Fifty-one (11.5%) were victims of violence and 393 (88.5%) experienced unintentional injuries. The VV patients were younger (40.10 vs 60.36; p<0.0001), and more predominantly male (92.16% vs 57.51%; p<0.0001). Total injury severity score (ISS), critical care length of stay (LOS), and total LOS were similar. VV patients were more likely discharged home (70.59% vs 55.47%; p=0.0403). They were significantly more likely to return to an emergency department (47.06% vs 23.16%; p<0.0005) and had more total number of ED visits per patient. Readmission rates, however, were not different (21.57% vs 16.28%; p=NS). The VV patients more frequently were underinsured (72.5%, vs 20.6%, p<0.005).

Discussion

Victims of violence presented to the ED significantly more often, despite similar injury scores, LOS, and being of younger age. Of these patients, only 26.2% of ED presentations resulted in readmission, suggesting the majority of patient complaints may have been able to be managed in an office-based setting. VV had significantly more underinsured or subsidized patients. Victims of violence are vulnerable and may benefit from more resources provided in the early post-discharge period.

## Introduction

Unplanned post-discharge events among trauma patients can result in significant increases in cost, morbidity, and even mortality [1-3]. The diversity of trauma patients in regard to age, comorbidities, and post-injury rehabilitation needs can make their post-acute management challenging. Thus, the rate at which trauma patients return to an emergency department (ED) soon after discharge remains high. Prior investigations have identified that up to 14% of injured patients return to an ED within 30 days of their discharge [4-8]. The accuracy of this data is in question, as many patients do not return to the index hospital [5]. Also, these investigations are retrospective in nature and no standardized prospective capture method currently exists [5-7]. The American College of Surgeons Trauma Quality Improvement Program (TQIP) aggregates injury data from over 825 trauma centers globally and provides participating programs with benchmarks for comparison [8, 9]. Although, TQIP has become a valuable tool, it has limited data regarding readmissions and post-discharge emergency department visits are not captured. Our group has previously deployed a modified ACS-TQIP (MTQIP) methodology to retrospectively review post-acute events in trauma patients. This investigation identified significantly higher readmission rates compared to the standard methods documented in trauma registries [8]. The current review expands upon the modified methodology to investigate post-trauma events prospectively, akin to the ACS National Surgical Quality Improvement Program technique.

Improving the capture of post-discharge events may allow trauma programs to better prepare their most vulnerable populations for discharge. Trauma patients of all mechanisms frequently present to the ED post-discharge, often for non-urgent reasons [5-7, 10, 11]. Penetrating trauma has been associated with an increased rate of post-discharge ED utilization compared to blunt mechanisms [5]. Given that most penetrating trauma stems from violent crime it stands to reason those victims who suffer injuries because of intentional violence represent a particularly vulnerable population. Their injuries as well as socioeconomic status may put them at risk for multiple post-hospitalization complications. It is known that trauma recidivism is high in this population, with a five-year re-injury rate of 50% and nearly 20% will ultimately die from violence-related injuries [1, 2]. We hypothesized that victims of violence represent a high-risk population for post-trauma ED resource utilization, and may benefit from focused efforts to provide early post-discharge follow-up. Utilizing our MTQIP process we sought to better characterize issues that victims of intentional violence face after discharge.

## Materials and methods

Saint Francis Hospital is a 617-bed, ACS verified Level-One trauma center located in Hartford, Connecticut. Institutional Review Board (IRB) and scientific review committee approvals were obtained. From January 1st, 2019 to December 31st, 2019 all injured patients at least 18 years old were prospectively reviewed. Those who were evaluated by the trauma service and warranted an inpatient admission for their injuries were included.

Patients were recruited as subjects during their index trauma admission. Informed consent was obtained as was reliable contact information and preferred contact method. The project, as well as follow-up questionnaire, was explained to the patient and with family representation when available. Patient demographics including age, gender, and socioeconomic data, and trauma-related metrics including mechanisms of injury, abbreviated injury scores (AIS), injury severity score (ISS), critical care length of stay (ICULOS), and total LOS were collected. Thirty days after discharge, patients were contacted and interviewed for discharge disposition, unplanned hospital or emergency department visits, admissions, and any interventions or procedures experienced since discharge. Local electronic medical records were reviewed for available details and documented post-acute events.

Patients were categorized into two groups, victims of violence (VV) and unintentional trauma (UT). VV included all forms of assault (gunfire, stabbings, and other physical assault). The UT group encompassed all other forms of trauma (falls, motor vehicle crashes, pedestrians struck, industrial injuries, etc.). Indications for post-acute presentations were compared between medical records and self-reports and were divided into broad categories including non-trauma-related medical concerns, trauma-related pain, wound/infection concerns, repeat trauma events, drain issues, suture or staple removal, and additional trauma-related complaints. Consultation to the trauma service was also reviewed for patients who returned to our hospital’s ED. The primary payer for each patient’s index trauma admission was collected. Underinsured was defined as those with Medicare, Medicaid or no health insurance at the time of their index trauma admission. Simple comparative statistics, Fisher's exact test, Student's t-test, and chi-square analysis were used to determine statistical significance, with a significance set at p = 0.05. Data was collected using Microsoft Excel (Microsoft® Corp., Redmond, WA) and analyzed using Statistical Package for Social Sciences (SPSS Statistics Version 24, IBM Corp., Armonk, NY).

## Results

During the study period, 2096 patients were evaluated in the emergency department for traumatic injury, and of them, 883 were admitted to an inpatient stay for their injuries. Of these patients, 444 (50.3%) consented to the study and were enrolled. Among the recruited subject population, 51 (11.5%) were victims of violence, and 393 (88.5%) experienced unintentional trauma. Telephonic follow-up was achieved in 340 patients (76.6%; 42/51 (82.4%) of VV and 298/393 (75.82%) of UT; p=NS). VV patients were significantly younger (40.1 years vs 60.4 years; p<0.0001), and more predominantly male (92.2% vs 57.5%; p<0.0001).

VV patients had a lower abbreviated injury score (AIS) for head/neck (0.37 [0.07] vs 1.03 [0.14]; p=0.0014), and higher AIS for face (0.22 [0.08] vs 0.17 [0.02], p=0.008) and abdomen (0.84 [0.17] vs 0.49 [0.05], p=0.035). Other AIS defined regions did not differ, nor did the average total ISS (8.59 [0.87] vs 9.13 [0.33]; p=NS). In regards to the inpatient admission, critical care LOS and overall LOS did not differ between groups (Table 1). At discharge, VV were more likely to be transitioned home after admission than UT patients (70.59% vs 55.47%; p=0.040) whereas UT patients were more often transferred to rehabilitation or skilled nursing facility.

**Table 1:** Demographics and hospital factors SD: Standard deviation; AIS: Abbreviated injury scale; ISS: Injury severity score; LOS: Length of stay; ED: Emergency department

Variables	Victims of Violence (n=51)	Unintentional Trauma (n=393)	p
Age, mean (SD)	40.10 (15.52)	60.36 (20.68)	<0.0001
Male	92.16%	57.51%	<0.0001
AIS			
-Head/Neck	0.37 [0.14]	1.03 [0.07]	<0.0001
-Face	0.22 [0.08]	0.17 [0.02]	0.008
-Chest	0.72 [0.18]	0.89 [0.07]	NS
-Abdomen	0.82 [0.20]	0.49 [0.05]	0.035
-Extremity	0.84 [0.17]	0.85 [0.06]	NS
-External	0.04 [0.04]	0.06 [0.03]	NS
Total ISS	8.59 [0.87]	9.13 [0.33]	NS
Critical care LOS in days (SD)	0.78 (1.76)	1.12 (3.20)	NS
Total LOS in days (SD)	4.92 (4.80)	4.13 (4.09)	NS
Disposition to home (%)	36 (70.59)	218 (55.47)	<0.04
ED visits, patients (%)	24 (47.06)	91 (23.16)	<0.001
ED visits, total (visits per patient)	42 (1.75)	112 (1.23)	<0.002
Overall readmission rate (%)	11/51 (21.6)	64/393 (16.28)	NS
Readmission rate per ED Visit (%)	11/42 (26.2)	64/112 (57.1)	<0.001
Underinsured (%)	37 (72.5)	81 (20.6)	<0.0001

Victims of violence were significantly more likely to return to an emergency department within 30 days of discharge (VV 24/51 (47.1%) vs UT 91/393 (23.2%), p<0.001). VV patients were also more likely to make multiple visits in the early post-discharge period, with 24 patients accounting for 42 visits vs 91 patients making 112 visits in the UT group (1.75 visits per patient vs 1.23, p=0.002). Visits to non-index institutions were captured as well, but visit frequency did not differ. Overall readmission rates did not differ between groups, (11/51 (21.6%) vs 64/393 (16.3%), p=NS). However, readmission rates per ED visit were significantly lower in the VV group at 11/42 (26.2%) vs 64/112 (57.1%), respectively (p<0.005). VV patients also had fewer post-acute admissions to non-index institutions compared to in the UT group (0/42 (0%) vs. 8/64 (12.5%), p=0.018). Evaluation of the primary payer for the index trauma admission found VV patients were more likely to be underinsured compared to the UT group with 37/51 (72.6%) vs 81/393 (20.6%); p<0.005.

## Discussion

Our prospective review demonstrated that victims of violence returned to the ED significantly more often than those suffering unintentional trauma. Previous investigations identify advanced age, comorbid conditions, higher injury severity, and disposition to a facility as the major contributors to readmissions [10-14]. However, our study conflicts that with younger patients who were discharged home were more likely to return to the hospital. While differences existed in injury severity to head/neck, face, and abdominal regions between groups, it is unclear if those differences contributed as the total ISS was similar. Furthermore, the average ISS between the groups was approximately 9 which does not represent a critically injured patient. This suggests patient-related factors as opposed to injury-related factors as the root cause of their post-discharge events. There was also no difference in ICULOS and total hospital LOS, again pointing to patient-related as opposed to injury-related factors.

VV patients had significantly higher rates of discharge to home, rather than a rehabilitation facility. The exact etiology of this discrepancy may be multifactorial. The VV was significantly younger compared to a high number of the UT group consisting of elderly falls (Table 2), thus making their discharge to a rehabilitation or extended care facility more likely. Although the correlation between disposition and underinsured status, making placement in acute rehabilitation facilities more challenging, cannot be overstated. Other socioeconomic factors may be involved, diminishing healthcare access in the VV population despite similar levels of injury.

**Table 2:** Mechanism of injury

Mechanism	n
Victims of Violence	51
- Gunshot wounds	- 20
- Stab wounds	- 15
- Assaults	- 16
Unintentional Trauma	393
- Fall from standing/sitting	- 142
- Fall from height/stairs	- 77
- Motor vehicle crash	- 97
- Motorcycle crash	- 32
- Recreational/industrial	- 22
- Pedestrian struck	- 17

Despite the higher rates of ED visits among our VV population, the readmissions rates overall did not differ between the groups. Furthermore, the VV group had a higher rate of single patients that made multiple ED visits within 30 days of their discharge. The most frequent indication for return in the VV group was trauma-related pain, however, many of the other presentations were for less-urgent indications such as wound/drain concerns or removal of stitches/staples (Table 3). In contrast, the UT group was much more likely to return for other non-trauma-related medical complaints, likely given their more advanced age and comorbidities. The number of wound or drain concerns resulting in an ED visit were much less frequent in the UT group (Table 3). The resources provided in the post-discharge period likely contributed to this discrepancy. UT patients with resources such as rehabilitation facilities to provide wound care and assessments of a patient’s pain may have prevented unnecessary ED visits. If issues arise they may also be more likely to direct their care through an office-based setting as opposed to the ED. Whereas, the VV who were more likely to be home, as well as less likely to have prompt access to medical care seem to have directed their concerns through the ED. This is further supported by the fact that although the rates of ED visits were higher in the VV group, only around one-quarter of those visits resulted in a readmission. This readmission rate was much lower compared to the UT group where over half of their ED visits resulted in a readmission. Given the significant discrepancy between the groups in regards to their insurance status, post-discharge resources seem to be a contributing factor to early ED visits.

**Table 3:** Emergency department visit chief complaints

Variables	Victims of Violence (n=42)	Unintentional Trauma (n=112)	p
Other medical complaints (%)	5 (11.90)	57 (50.89)	<0.0001
Repeat trauma events (%)	3 (7.14)	18 (16.07)	NS
Trauma-related complaints (%)	3 (7.14)	15 (13.39)	NS
Wound/Infection concerns (%)	11 (26.19)	11 (9.82)	0.01
Trauma-related pain (%)	15 (35.71)	10 (8.93)	0.0001
Stiches/Staple removal (%)	3 (7.14)	1 (0.89)	0.0303
Drain concerns (%)	2 (4.76)	0 (0.00)	0.025

Post-acute emergency department visits have been examined in trauma previously, but mostly in retrospective reviews [5, 6, 14-18]. Abou-Hanna et al. reported a 14% return to ED rate in 30 days post-injury, citing penetrating trauma was a risk factor [5]. Our prospective study corroborates this finding, albeit at a much higher rate, and also adds other forms of intentional injury. Our data also captures more events by prospectively tracking patients soon after discharge. Our questionnaire not only identifies representations but also captures the chief complaint for that presentation, associated readmissions, and further procedures. The MTQIP system also captures those who presented to outside institutions that would go unrecognized by standard processes of other trauma registries. By identifying all assaults, not just penetrating trauma, we further highlighted that patient and socioeconomic factors, as opposed to just mechanism, are a major contributor to early post-discharge ED utilization.

Our investigation had several limitations. It is well established that some patients utilize the ED for primary care due to socioeconomic factors. We did not capture if any of our captured patients utilized the ED for primary care prior to their traumatic injury admission. We also did not capture what each individual patient's post-discharge resource needs were, such as if they had drains, wounds, catheters, etc. So the resources needed, what was available to them and if that contributed to early post-discharge events is unclear. However, it is standard at our institution to involve our physical therapy and case management teams in all trauma admissions to help identify post-discharge needs. It is also standard to provide follow-up information for the trauma surgery clinic as well as any consulting (both medical and surgical) services that were involved in the patients care. Data from post-discharge events was also self-reported, and for those who visited hospitals outside our system, we did not have medical record details readily available. Data relied on patient reports who may lack detailed medical knowledge. Additionally, 142 patients (31.98%) were not reached telephonically. Of these, 38 had ED visits documented in our system. It remains unclear how many of these patients had additional post-discharge events at outside institutions. The diversity in our population also creates limitations in comparing the groups. The VV group being significantly younger compared to the UT population may have contributed to them receiving less post-discharge resources as they may have been more independent at discharge. Although their higher rates or representation demonstrates that this group would benefit from more resources after discharge, even if those resources are recovery education based. The UT group also had many ED visits for non-trauma-related medical complaints. Whether these presentations had any relation to their prior injury or hospitalization and if they were avoidable is unclear.

The opportunity to better prepare our VV patients for post-discharge needs is apparent. This vulnerable group faces a multitude of physical and psychological challenges after their injuries [19-21]. While enhanced recovery processes are challenging in the urgent or emergent trauma patient, there may be opportunities to improve post-trauma recovery with targeted education. This project has led our trauma service to incorporate the 30-day post-discharge phone call into our standard practice for all patients. Our team is also more aware of these post-discharge events and provides additional education at the time of discharge to patients identified as being higher risk for post-acute events. This includes shorter interval follow-up and further emphasis on post-discharge instructions regarding who to call if issues arise.

Victims of violence are vulnerable and may benefit from more resources provided in the early post-discharge period. Better post-discharge monitoring of these patients with direct contact and health information exchanges is warranted. Detailed chart review and post-discharge phone calls enhanced our reporting of these events, allowing providers to find solutions to mitigate them.

## Conclusions

A modified trauma quality improvement program including post-discharge phone calls improved our documentation of early post-discharge events. Victims of violence had significantly higher rates of early ED utilization compared to patients experiencing unintentional injury. Patients among those victims of violence were also younger and were more likely to be underinsured. We identified VV as a population that warrants more resources in the early post-discharge period to prevent unnecessary ED visits, and potentially improved recovery.
